# Support received by family members before, at and after an ill person’s death

**DOI:** 10.1186/s12904-021-00800-8

**Published:** 2021-06-24

**Authors:** Anna O’Sullivan, Anette Alvariza, Joakim Öhlén, Cecilia Larsdotter

**Affiliations:** 1 Palliative Research Centre, Department of Healthcare Sciences, Ersta Sköndal Bräcke University College, Stockholm, Sweden; 2 Capio Palliative Care, Dalen Hospital, Stockholm, Sweden; 3 Centre for Person-Centered Care, University of Gothenburg, Gothenburg, Sweden; 4 Institute of Health and Care Sciences, Sahlgrenska Academy At the University of Gothenburg, Gothenburg, Sweden; 5 Palliative Centre, Sahlgrenska University Hospital Västra Götaland Region, Gothenburg, Sweden; 6 Department of Nursing Science, Sophiahemmet University, P.O. Box 5605, 114 86 Stockholm, Sweden

**Keywords:** Family members, Support, Information, Communication, Palliative care, End-of-life care

## Abstract

**Background:**

It is widely recognised, that family members are central to care of people with advanced illness, and that support should be provided to all family members in need thereof. The aim of this study was to investigate family members’ experiences of support received during the last three months of life, at the time of death and after the death of a person with advanced illness.

**Methods:**

A retrospective cross-sectional survey design was employed, using the VOICES(SF) questionnaire and multiple methods for data analyses. The sample consisted of 485 bereaved family members (aged: 20–90 years old, 70% women) of people who died in hospital between August 2016-April 2017.

**Results:**

Of the family members, 58,8% reported they had received enough help and support during the illness, whereas 30,2% had not. Family members’ comments about support during the illness were mainly related to care the ill person had or had not received, rather than about support they themselves received. Of all family members, 52,8% reported having had enough support at the time of the ill person’s death. Related to support at death, 14,6% reported that the imminence of death was not clear, which was described as having affected their opportunity to be with the dying person at the time of death. Of all, 25,2% had a follow-up conversation after the death, 48% did not and did not want to, and 21% had no follow-up conversation, but would have liked one. A follow-up conversation was described as helpful for the bereavement process, and disappointment was expressed when not receiving support after the death.

**Conclusions:**

Family members’ experiences of support were partly related to whether the ill person’s care needs were fulfilled. Healthcare staff expressing empathy and respect in the care of dying people and their family members were important for family members’ experiences of support. Family members’ difficulty recognising that death was imminent and the importance of healthcare staff providing them with clear information were expressed in connection with support at death. Follow-up conversations were valued by family members, especially if with a healthcare professional who was present at the time of death.

## Background

It is widely recognised that family members are often central to care of people with advanced illness. During the illness period and after death, care and support should be provided to patients as well as their family members. Being a family member of a person with advanced illness can have a multidimensional impact: psychological, with increased stress and worry; physical, due to various —at times burdensome— practical care activities; social, involving limitations on social life; and financial, having to take time off work to be a caregiver and/or the absence of income for the ill person [1, 2]. Hence, support for family members is an essential part of palliative care.

Several previous studies have pointed out different types of support for family members. Psychological and emotional support is often received through some form of counselling, either individually or in groups. Practical support can involve, for example, help with household chores or respite for the family member, if the ill person is living at home. Family members’ support needs vary and may fluctuate during the illness period and after the death of the ill person [3, 4]. International studies have shown that the support family members do or do not receive may affect their health —for example, in terms of anxiety and depression symptoms— which may result in a worse bereavement experience [5–7].

Various barriers for adequate support to family members have been found, such as healthcare staff feeling unprepared or lacking time to communicate. Another barrier for providing adequate support is family members and the ill person not accepting or grasping the prognosis [8, 9]. In Sweden, support available to family members of people with advanced illness varies — depending on, for example, the care setting and geographic area of residence [3, 10]. There is a sparsity of studies that investigate overall support family members received during the last period of illness of a person, at death and throughout the period after death — and it is these issues that this study aims to explore.

## Methods

### Aim

The aim of this study was to investigate family members’ experiences of support received during the last three months of life, at the time of death and after the death of a person with advanced illness.

### Design

This study employed a retrospective cross-sectional survey design using The VOICES (SF) (Views of Informal Carers – Evaluation of Services) (Short Form) questionnaire, and multiple methods for data analyses.

### Setting and sample

The study sample consisted of adult bereaved family members of persons with advanced illness, who died in four hospitals, located in two Swedish healthcare regions, between August 2016 and April 2017. In Sweden, care at the end of life can be provided at home, in hospitals, in nursing homes and in specialized palliative care units, e.g., hospices. The deceased persons had all died in hospital but had received care in several care places and settings, in one of the healthcare regions. The hospitals were used as recruitment settings since 42% of the Swedish population die in hospital [11].

The inclusion criteria were that the deceased persons had identifiable bereaved family members; both deceased persons and bereaved family members needed to be 18 years old or older; and the deceased person had to have died from underlying causes of death (ICD-10 codes) in accordance with the Murtagh et al. [12] model: HIV/Aids; Malignant Neoplasms (Cancer); Alzheimer’s disease, dementia and senility; Neurodegenerative diseases; Heart diseases including cerebrovascular diseases; Respiratory diseases; Liver diseases and Renal diseases.

The questionnaires were sent to the bereaved family members four to twelve months after the death of the deceased person. This is in accordance with experiences from previous studies using VOICES (SF), in which it was concluded after testing different time frames, that 4–12 months after death was an appropriate time period. It is a fine balance between intrusion in the grieving process and avoiding the likely gradually reduction in the ability to recall over time [13–17].

### The VOICES (SF) questionnaire

The VOICES (SF) is a questionnaire that retrospectively evaluates the quality of care received in several different care places, during an ill person’s last three months of life, based on bereaved family members’ reports. The full version of VOICES (SF) has been translated and validated into other languages by other research groups [18–20]. The Swedish version [21] of the questionnaire is divided into domains such as Care at home; Care homes; Hospital care; and Specialized palliative care units/hospice care. It contains 74 items, firstly about characteristics (e.g., age, sex, educational attainment, relationship to the deceased person), followed by items about for example symptom relief, communication, involvement in decision-making, being treated with respect and dignity by care staff and satisfaction with care reported for different care places/care services. The questionnaire includes structured items about help and support received during the illness, at the death of the ill person and after the death. Additionally there are open-ended questions — some in connection with the items about support and help, and three open-ended questions at the end of the questionnaire. There have been previous studies using VOICES in different patient groups and in various healthcare settings, both in cross-sectional studies and at a population level, mainly in the United Kingdom, where the questionnaire was developed [13, 17].

### Study variables

Variables used to describe the characteristics of the bereaved family members were age, sex and educational level. The same variables were used for the deceased persons, with additional ones also included — namely time of illness before death, diagnosis, care place/care provider, number of care places and the relationship between the deceased person and their family member.

The variables chosen for this study were questionnaire items about the following: support during the illness; having been contacted in time to be present at the death; support from the staff at the time of death; being treated with respect by staff at the time of death; and support after the death. Five open-ended questions were also included. Of these, two were comments linked to the items about support during the illness and being treated with respect by staff at the time of death. The other three were open-ended questions in the final part of the questionnaire; one asking if the respondent would like to add anything else about the care and support received, and two questions asking whether anything was good or bad about the care (Table 1).

**Table 1:** Study variables and analyses

**Items**	**Response alternatives**	**Analysis**
Overall, do you feel that you and your family got as much help and support from health and social services as you needed when caring for him/her?	- Yes, we got as much support as we needed/yes - We got some support, but not as much as we wanted - No, although we tried to get more help - No, but we did not try to get more help - We did not need help	Quantitative descriptive
Please feel free to make comments in the space below	Open-ended question	Qualitative – interpretive description
Were you contacted soon enough to give you time to be with him/her before he/she died?	- Yes - No - I was already there - It was not clear he/she was going to die soon - I couldn’t have got there anyway - I was not contacted	Quantitative descriptive
Were you or his/her family given enough help and support by the healthcare team at the actual time of his/her death?	- Yes, definitely - Yes, to some extent - No, not at all - Don’t know	Quantitative descriptive
Were you or his/her family treated with respect by the staff after he/she had died?	- Yes - No - Don’t know - Does not apply, I had no contact with the staff	Quantitative descriptive
Please feel free to make comments in the space below	Open-ended question	Qualitative – interpretive description
Since he/she died, have you talked to anyone from health and social services, or from a bereavement service, about your feelings about his/her illness and death?	- Yes - No, but I would have liked to - No, but I did not want to anyway - Unsure	Quantitative descriptive
Please use the space below if there is anything else you would like to tell us about the care and support you received	Open-ended question	Qualitative – interpretive description
What, if anything, was good about the care?	Open-ended question	Qualitative – interpretive description
What, if anything, was bad about the care?	Open-ended question	Qualitative – interpretive description

### Recruitment and data collection

Of all the patients who died in the recruitment hospitals during the study period, 78% (*n* = 1277) were eligible for inclusion. Based on the inclusion criteria, hospital administrators identified the deceased persons, and their bereaved family members were identified via the hospital’s patient records by one healthcare professional at each hospital (assigned to assist Author 1). Postal addresses of the bereaved family members were retrieved from publicly available databases. Written information about the study was sent, including contact information for one of the researchers (Author 1), information stating that the study was performed in cooperation with the hospital in which their family member had died and the VOICES (SF) questionnaire along with a pre-paid return envelope. The written information assured confidentiality and the right to withdraw from the study at any time without explanation. Consent was considered to have been obtained upon return of a questionnaire; no other written informed consent for participation in the study was obtained. To be sensitive towards the family members who may be considered vulnerable due to bereavement and may not wish to participate, no reminders were sent.

### Characteristics of the deceased persons and their bereaved family members

The deceased persons were between 40 and 90 years or older (64% were 80 or older) and 50,3% were men. Of the deceased persons, 72,5% had lower secondary education, 11,1% higher secondary education and 15,5% higher education. The most common underlying cause of death was heart diseases, including cerebrovascular diseases (56,3%), followed by cancers (15,8%) and respiratory diseases (15,1%) (Table 2). The participating family members were between 18 and 90 years old or older and 70,7% were women. Of the family members, 29,5% had lower secondary education, 30,5% higher secondary education and 39,4% higher education. About half (51,8%) were children of the deceased person and 34,5% were spouses or partners (Table 2). Of the deceased persons, 79,2% had been cared for at home at some point during the last three months of life, with 52% receiving care from general practitioners (GPs); 17,9% from specialised palliative home care and 36,7% received care from district- and county nurses. Furthermore, 90,7% had received hospital care, 27,4% nursing home care and 15,7% care in a specialised palliative care unit. The number of places of care during the last three months of life ranged from 1 to 4, with two places being most common (63,4%), followed by three places (20,4%), one place (12,9%) and, least commonly, four places of care (3,3%).

**Table 2:** Characteristics of the deceased persons and their family members

	Deceased persons		Family members	
	%^a^	n	%^a^	n
Sex (missing = 0/0)^b^				
Male	50.3	(244)	29.3	(142)
Female	49.7	(241)	70.7	(343)
Age (missing = 1/8)^b^				
18–39			2.4	(12)
40–59	3.5	(17)	29.1	(141)
60–69	8.9	(43)	31.3	(152)
70–79	23.1	(112)	22.9	(111)
80–89	36.7	(178)	11.3	(55)
90 +	27.6	(134)	1.2	(6)
Educational attainment (Missing = 5/3)^b^				
Lower secondary education	72.4	(351)	29.5	(143)
Higher secondary education	11.1	(54)	30.5	(148)
Higher education	15.5	(75)	39.4	(191)
Underlying cause of death 1^c^				
Cognitive diseases	1.0	(4)		
Neurodegenerative diseases	1.0	(4)		
Liver diseases	1.5	(6)		
Renal diseases	9.4	(38)		
Respiratory diseases	15.1	(61)		
Cancer	15.8	(64)		
Heart diseases (incl. cerebrovasular)	56.3	(228)		
Underlying cause of death 2^c^				
HIV/Aids	0.3	(1)		
Liver diseases	0.9	(3)		
Neurodegenerative diseases	1.2	(4)		
Cognitive diseases	3.5	(12)		
Renal diseases	4.4	(15)		
Respiratory diseases	10.2	(35)		
Cancer	15.2	(52)		
Heart diseases (incl. cerebrovasular)	64.3	(220)		
Length of illness before death (Missing = 6)^b^				
Sudden death	5.4	(26)		
< 24 h	2.1	(10)		
24 h – 1 week	10.7	(52)		
1 week – 1 month	13.0	(63)		
1 month – 6 months	14.8	(72)		
6 months – 1 year	10.3	(50)		
1 year or more	42.5	(206)		
Relationship (missing = 4)^b^				
Spouse			34.5	(166)
Child			51.8	(249)
Other^d^			13.7	(66)

^a^ Column percentage displayed

^b^ Missing = 0/0 shows the number of missing cases for deceased persons/bereaved family members

^c^ Underlying causes of death according to Murtagh’s (2014) model for potential palliative care needs

^d^ E.g., parent, sibling, friend

The characteristics of the bereaved family members who chose to answer one or more of the open-ended questions differed slightly from the total sample regarding sex (77,7% women), educational attainment (52,2% higher education and 19,3% lower education) and the deceased person’s educational attainment (20% higher education and 67,4% lower education).

### Response rate

The response rate was 37,9%, resulting in a total of 485 bereaved family members participating in the study. The non-responding family members’ individual characteristics (e.g., age, sex, educational attainment) were not available. The deceased persons’ profiles linked to the non-responders did not differ from the sample.

### Analysis

Both statistical and qualitative methods were used for data analyses (Table 1). Initially, the quantitative data was analysed, after which the open-ended responses were analysed qualitatively to deepen the understanding of the family members’ responses to the items about support received.

#### Statistical analyses

Descriptive statistical analyses were used to explore the variables derived from the questionnaire items and characteristics of the deceased persons and their family members. For statistical computations, Statistical Package for the Social Sciences (SPSS) version 21.0 (IBM Corp., Armonk, NY, USA) was used.

#### Qualitative analysis

**Fig. 1:**
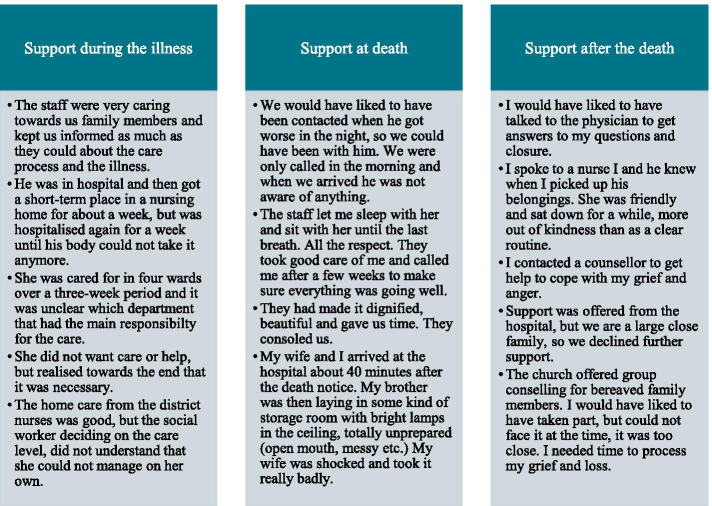
Exemplary quotations of bereaved family members’ comments about support in the open-ended questions

On average, the responses were 1 or 2 sentences long, ranging from a couple of words to full pages (see Fig. 1 below for exemplar quotations). Moreover, there was a variety in content; some were quite exhaustive stories, while others were shorter substantive responses. There was in total 638 responses by 270 family members to the five open-ended questions, divided as follows: Help and support during the illness (n94); Being treated with respect by staff at the time of death (n75); Anything else you would like to tell us about the care and support you received (n116); Was anything particularly good about the care (n184); Was anything particularly bad about the care (n169). After excluding responses not related to the study aim, 529 responses (20 full pages of single-spaced text) were analysed. The analysis was guided by interpretive description, which is a viable approach for generating knowledge applicable to clinical practice [22].

Initially, all the open-ended responses were read to obtain an overall picture, then they were read again in the context of the family members’ varied responses to the items about support, to discover patterns and deepen the understanding of the quantitative responses. Thereafter, the text was broadly coded, identifying meanings and variations of contextual descriptions of support, which were organised into patterns of experiences. These were interpreted through a process of asking questions such as “what is the underlying meaning of this?” The descriptions and interpretations of the text were continuously discussed and revised within the research group for clarification and further development of the analysis.

## Results

Family members’ experiences of support received —during the illness, at death and after the death of a person— were varied. The results are presented under three headings: help and support during the illness period; circumstances and support at the time of death; and follow-up conversation and support received after death.

### Help and support during the illness period

Of the bereaved family members, more than half (58,8%) reported that they got as much help and support as they wanted during the illness period. However, about a third reported that they did not get enough help and support (Table 3).

**Table 3:** Overall, do you feel that you and your family got as much help and support from the health and social services as you needed when caring for him/her?

Response	N	%
Yes, we got as much support as we wanted	285	58.8
Yes, we got some support, but not as much as we wanted	91	18.8
No, although we tried to get more help	27	5.6
No, but we did not ask for more help	28	5.8
We did not need any help	33	6.8
Missing	21	4.3

#### Being supported related to the fulfilment of care needs

Family members’ reports rarely mentioned experiences of actual help and support they themselves received during the illness period. Instead, many family members had written about the care that the ill person did or did not receive. Whether care needs were fulfilled or not seemed to have been the main focus for family members’ reports regarding experiences of help and support they received during the illness period. For example, family members described how the ill person had been denied admission to a nursing home or had been discharged without a care plan after an emergency visit to the hospital. This was described as a disappointment with healthcare and the process of decision-making process regarding care level needs. One family member wrote:

My 91-year-old father-in-law needed help with pretty much everything. He could walk a few steps with a walker indoors but had repeatedly fallen and had to go to hospital urgently. In January, an application for a place in a care home was sent, but it was rejected on the grounds that he could get the help he needed at home... Had he been given a place and given help and supervision, then he would probably not have died in February. He died after a fall at home. [Daughter-in-law]

Furthermore, family members described lack of care or insufficient care, which they related to frequent emergency visits with long waiting times. An illustrating example was when the ill person had been cared for at home without the right level of care and supervision:

There were many hospital admissions in the last year when his COPD deteriorated. It was pretty disruptive; he was often sent back home. The preparedness in emergency situations was good. I had wished for something in between shorter hospital stays and sent home to no care at all. More home care and follow-up at home. [Son]

Family members wrote about the importance of professionals in the position of deciding on care level for the ill person also listening to the family members’ views on care needs. For example, care was sometimes not provided since the ill person did not want care, even though the family member thought that it was needed: “My mother did not want help but was in great need of it.” [Daughter] This created stress for the family member who could not be close at hand all the time. This was especially stressful when the ill person lived alone and had a history of many fall injuries or a dementia diagnosis. A daughter summarised this situation, outlining: “… the difficulty and complexity of being a family member balanced with the ‘personal integrity’ of the patient*.*”

Furthermore, family members described how they had to act as co-ordinator for the ill person’s care, due to lack of communication and cooperation between health care providers. Family members described feeling as if they had to make sure that things were being done. This raised questions about who actually had the primary responsibility for care — the healthcare providers, the patient or the family members. A daughter wrote:

The coordination, communication, responsibility and feedback from homecare services that have no idea who has been or what has been done during the weekends... The outsourced care at the weekend cannot be contacted. When they call me, they do not know what medicine my demented mother should have!! And you never met the same staff. [Daughter]

### Circumstances and support at the time of death

#### Being contacted in time

Of the bereaved family members, 49,5% reported that they had been contacted in time to be with the ill person at death. For 7,8% of the family members, this was not the case, and for another 14,6% it was not clear how soon death would come (Table 4).

**Table 4:** Were you contacted soon enough to give you time to be with him/her before he/she died?

Response	N	%
Yes	240	49.5
No^a^	38	7.8
I was already there	88	18.1
It was not clear that he/she was going to die soon	71	14.6
I could not have come anyway	16	3.3
I was not contacted^a^	14	2.9
Missing	18	3.6

^a^The response “No” means that the family member was contacted, but not soon enough to be with the ill person at death. “I was not contacted” means that the family member was not contacted at all

Family members described that they had appreciated the opportunity to say a last goodbye. They were also grateful that the ill person did not have to die without any family present. Not having been informed about the ill person’s deterioration and, hence missing the opportunity to be there was described as upsetting: “He died in the dining room, no one was with him, he had been unwell…no one knew when he died”. [Wife]

Family members also reported how difficult it was to understand when the end would come; how they had not realised how close death was and were not prepared for it to come as soon as it did: “The end was near but I did not realise”. [Niece]

#### Being supported and treated with respect by staff at the time of death

**Fig. 2:**
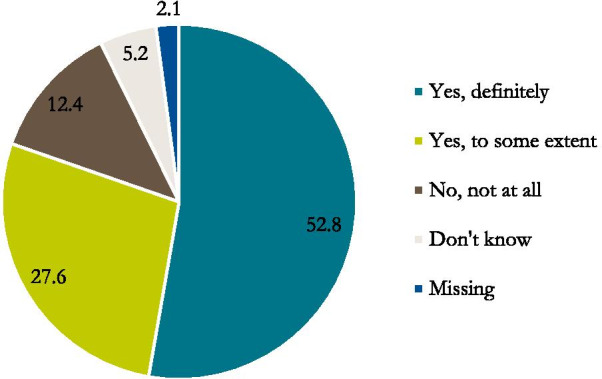
Percentage of family members responding they or the ill person’s family got enough help and support by the healthcare team at the actual time of his/her death

**Fig. 3:**
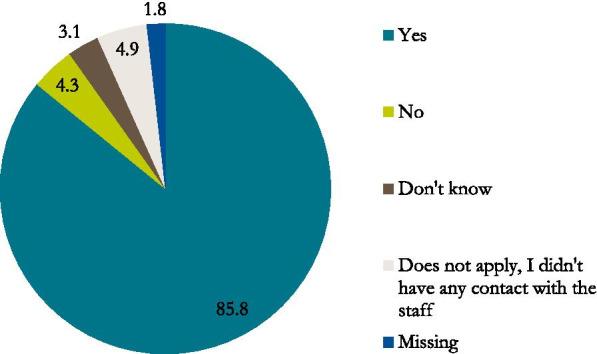
Percentages of family members responding that they were treated with respect by the staff at the time of the ill person’s death

At the time of the ill person’s death, approximately half (52,8%) of the bereaved family members reported that they had received enough help and support from the staff. Of the participants, 27,6% experienced having received help and support to some extent and another 12,4% reported ‘No, not at all’ with regard to getting enough help and support at the time of death (Fig. 2). The majority of family members (85,8%) reported that they had been treated with respect by the staff at the time of the death (Fig. 3).

Many comments were about the staff’s approach around the time of death. To have been given space and time to mourn, and to have been shown empathy—through expressing their condolences or a gesture like a hug in connection with the death, were described as supportive and respectful actions. In addition, family members appreciated staff showing respect by caring for the deceased person by cleaning, dressing and preparing the person and making the room look nice with flowers or lit candles: “They asked if we needed anything, such as sleeping pills, gave information about counselling and made the room nice with candlelight”. [Wife] However, some family members also described negative experiences and lack of support. For example, how staff mostly handled practical matters rather than showing empathy or expressing their condolences: “They asked if we wanted him autopsied”. [Friend] Other examples were family members only being told what forms to fill out and by what time they had to pack up the deceased person’s belongings and leave the room.

### Follow-up conversation and support received after death

After the death of the ill person, around a quarter (25,2%) of the family members had spoken to someone from healthcare, social services or bereavement services about their feelings. About a fifth (21,0%) had not spoken to any of these, but would have liked to do so, whereas 48% had not spoken to anyone and did not want to (Table 5).

**Table 5:** Since he/she died, have you spoken to anyone from healthcare, social services or bereavement services about your feelings about his/her illness and death?

Response	N	%
Yes	122	25.2
No, but I would have liked to	102	21.0
No, I didn’t want to anyway	233	48.0
Not sure	15	3.1
Missing	13	2.7

Family members expressed disappointment in not receiving support after the death. They reported for example disappointment in not being offered support at all or not being contacted despite having been told someone would call. Further examples were family members who had been given a number to call, but not got an answer when they called. Some described how they had spoken to a counsellor, a nurse, a physician, staff from homecare services or nursing home staff, and others reported having had support from a priest, the church or bereavement groups. A positive experience of a follow-up conversation with someone from healthcare after the death was described as helpful for the bereavement process:

They called after about 6–8 weeks to see how we, the siblings, were. Absolutely superb way of working!! We have had the opportunity to come and talk to the physician afterwards and got explained what we wondered about the death of our mother. [Daughter]

Family members described wishing to have had a conversation with a healthcare professional who was present at the death and who could have answered their questions. They also described having had a follow-up conversation, but with the ‘wrong’ person and had not got their questions answered, for example regarding the circumstances at the time of death: “I received a phone call from a nurse at the ward where my father passed away. When I asked how he was before we arrived at the hospital, I got no answer. It was not documented in his file according to the nurse”. [Daughter] This left the family members wondering and ruminating about how the ill person had been at the end.

## Discussion

In this study, the results show appreciative reports about the support family members received during the last three months of an ill person’s life, at the time of the death, and after the death. However, the results also show that the support was not optimal for all — about a third reported that they had not received enough help and support during the illness and only a quarter had a follow-up conversation after the death. Around half of the family members reported having had enough support at the time of the ill person’s death. In addition, the imminence of death was not clear for about 15% of family members, which was also described as having affected the opportunity for them to be with the dying person at the time of death.

The majority of family members reported that they received enough help and support during the deceased person’s illness — slightly higher than in a previous UK nationwide population study using the VOICES [13]. In the present study, family members’ reports about help and support received during the illness period seemed to have been focused on the fulfilment of the ill person’s care needs. An example was family members reporting feeling disappointed when the ill person was not granted a place in a nursing home or when the care at home was not considered to be sufficient. Unfulfilled care needs can burden family members who may have to step in to provide and co-ordinate care in the place of formal care.

Unfulfilled care needs were reported to have resulted in frequent visits to the hospital emergency room. There are several possible factors contributing to why the ill person did not receive the care needed. Firstly, it might be a question of care availability. In 2018, 40% of Sweden’s municipalities reported that they had a shortfall of nursing home beds [23]. In line with this, reports have shown that nursing home bed numbers have decreased by a quarter since the year 2000, without being replaced by increased homecare services. Instead, an increase in informal caregiving by family members or friends has been seen [2]. The percentage of the population in nursing home care in Sweden is comparable to other Scandinavian countries, such as Norway, Denmark and Finland, but high compared to countries in Southern Europe. The decrease in number of nursing home beds seen in Sweden and Norway can reflect a policy choice to move away from this form of care [24]. A move away from more institutionalised care can also be seen as reflecting the emphasis placed on achieving the WHO’s goal of ‘ageing at home’ [23]. Other contributing factors could be the difficulty in deciding on an adequate level of care and knowing in advance how continued care in the ill person’s home will work. For example, regarding discharge from hospital, it has been shown that several aspects are important, namely the discharge process [25, 26], the support available in the care place to which the person is discharged [27, 28] and follow-up after discharge [26, 29]. Care planning for ill people is also complicated by the different organisational structures and responsibilities of care providers. Care planning can be improved and re-admission to hospital prevented by the use of Advanced Care Planning (ACP) [28, 30]. Furthermore, the assessment of family members’ needs for support when caring for the ill person at discharge and at home after discharge may prevent the care at home from failing as well as re-admission to hospital [31].

In Sweden, home care and most forms of care for older people (including care in nursing homes) are regulated by the Social Services Act, and managed by the municipalities, whereas medical care needs are regulated by the Health and Medical Care Act and provided by different general or specialist care providers. This split in regulations and responsibility for care likely complicates the planning and delivery of care at the end of life. Further follow up by the municipality (through social services or home care services) and primary care after discharge could improve the discharge from hospital, and could also potentially prevent hospital re-admissions [25].

In the present study, family members reported care not being delivered, even though the family member thought it necessary, since the ill person did not wish to receive care. This was also shown in a study by Tarberg’s et al., in which family members described how they had to provide burdensome informal care, since the ill person did not accept formal care [32]. Diverse, contrasting views regarding care needs between the ill person, family members and staff can result in the ill person ending up with care that they do not want [25]. Differing views on the part of the ill person and family members about care needs could have been partly related to the ill person struggling with being at the end of life, in need of care and possibly trying to make sense of dying and accepting inevitable death [33, 34]. A way to support the ill person to grasp the situation and accept help is by healthcare staff supporting family members to initiate and strive for a more open communication about the illness, prognosis and individual wishes for the end-of-life care [35].

The importance of being informed of imminent death by healthcare has been previously reported, as has family members’ difficulty in seeing that the end is near [36, 37]. In the present study, family members reported that they wished healthcare staff had informed them that the ill person would die soon, to have given them a chance to be present. Family members not realising that the death was imminent could partly be due to them being unprepared for death and not informed enough to understand the process of dying. Furthermore, healthcare staff not being clear enough in their communication about death, by for example using euphemisms, like “her time is near”, could have contributed [36, 38, 39]. In line with this, previous research has shown that healthcare staff often feel uneasy discussing death and dying with patients and family members. There is also the difficulty for healthcare staff with regard to sharing information about a poor prognosis and imminent death if the ill person or family members are not open to receiving such information [37, 40]. Family members may need help interpreting information from healthcare staff, to appreciate how close death really is [36, 38, 39]. In this and previous studies, family members expressed the importance of, and gratefulness for, being able to spend the final moments with the dying person and the disappointment associated with not being able to do so [36, 39].

About half of the bereaved family members in this study reported that they got enough help and support from the staff at the time of the ill person’s death, which is slightly lower than reported in a UK national study [13]. Furthermore, around 12% of the family members reported not having received enough help and support at all at the time of the ill person’s death. Circumstances contributing to this could be related to family members’ reports of how they would have liked the staff to have shown more empathy and acknowledged their loss —through expressing their condolences or a gesture like a hug in connection with the death. The findings from previous research is in line with our findings; family members request more compassionate, sensitive and empathetic approaches on the part of healthcare staff at the end of life and at the time of death [5, 36, 41, 42].

A quarter of the family members had —by the time of study participation— spoken to someone from healthcare, social services or bereavement services about their feelings regarding the deceased person’s illness and death, and this was described as helpful in their grief. This is low compared to a nationwide UK study, in which twice as many bereaved family members had a follow-up conversation [13]. It is also low compared to the 67% of bereaved family members that were offered follow-up conversations, registered by healthcare providers in the Swedish Register for palliative care in 2019 [3, 43]. In the present study, a fifth of the family members had not spoken to anyone but would have liked to. Follow-up conversations may facilitate the bereavement process, and the lack of one may have the opposite effect [5, 7], with a risk of resulting in prolonged grief [44, 45]. In order to enable practical support for healthcare staff in their end-of-life communication, procedures related to follow-up conversations in the national guidelines for palliative care could be clarified. Including information about how, with whom and what topics that could be discussed. Hudson et al. [1] suggest that the family members should be contacted shortly after the death by someone from the healthcare team involved, to express condolences and set a plan for support according to needs, including a follow-up 3–6 weeks post death and again after 6 months. This however requires having routines for bereavement support and resources in place [1].

### Considerations and limitations

The response rate to the survey was rather low (37,9%) and could potentially have been improved by reminders and repeated mail outs. It was, however, an ethical choice not to do so, in order not to distress or put pressure on the bereaved family members. The response rate is in line with or higher than other studies using the VOICES (SF) [46–49]. Nevertheless, there was a good level of engagement from those who did participate; more than half (56%) chose to leave responses to the open-ended questions. Additionally, the quantity and quality of the qualitative data was considered sufficient to investigate the study aim. The interpretations and descriptions based on the open-ended responses were continuously discussed within the research group and these have been supplemented by participant’s quotations to confirm the interpretations. Views on the use of open-ended questions in surveys are mixed and so is the choice of analysis for these. Responses to open-ended questions can help to explain, illuminate or expand upon specific quantitative questions. However, methodological literature does not provide much guidance about ways of analysing open-ended question data [50]. The data about follow-up conversations and bereavement support does not provide insight into at what point after the death the family members had a conversation with someone from healthcare, about the content or with whom. There is no information on further bereavement support, except for the family members descriptions of such in the open-ended responses.

Of the participating family members in this study, 70,7% were women. The majority were children of the deceased persons, followed by spouses. In Sweden and in other countries, it is more common for women, than men, to provide informal care [2, 24, 51], hence the higher number of participating women in this study is probably representative for the population of informal carers. It is also worth noting that there were no deceased persons aged 18–39 years old (Table 2). Given the sample is based on hospital deaths and accidents are excluded, one probable assumption is that people aged 18–39 who die from prolonged and/or life-limiting illness (non-acute) are more likely to die at home or in hospices, than in hospitals. Previous research has shown that the likelihood of dying in hospital increases with age [11, 52]. Another consideration is that the whole sample consisted of people who had died in hospital. Hospital is the most common place of death in Sweden [11] and people who die in hospital commonly have several other places of care during the final stages of their illness trajectory, before dying in the hospital. The death in hospital and the care trajectory leading up to it, may however be reflected in the family members’ reports of the care. However, this study does provide new and important knowledge about the support family members received during a person’s illness, at death and after the death.

## Conclusions and implications

In this study, family members’ experiences of support were partly related to whether the ill person’s care needs were fulfilled. The study showed that healthcare staff expressing empathy and respect in the care of dying people and their family members were important for family members’ experiences of support. The family members’ difficulty recognising that death was imminent and the importance of healthcare staff providing them with clear information were both factors expressed in connection with family members’ experiences of support at death. Finally, the study showed that follow-up conversations were valued by family members, especially if with a healthcare professional who was present at the time of death. Clearer guidelines regarding end-of-life communication and support, as well as targeted training in palliative care and communication for healthcare staff, may improve family members’ experiences of support during a person’s illness, at the time of death and after death.

## Data Availability

The datasets generated and/or analysed during the current study are not publicly available, but are available from the corresponding author on reasonable request.
